# Access and utilisation of primary health care services comparing urban and rural areas of Riyadh Providence, Kingdom of Saudi Arabia

**DOI:** 10.1186/s12913-017-1983-z

**Published:** 2017-02-02

**Authors:** Ghadah Alfaqeeh, Erica J. Cook, Gurch Randhawa, Nasreen Ali

**Affiliations:** 1 Ministry of Health, Riyadh, Kingdom of Saudi Arabia; 2 Department of Psychology, University of Bedfordshire, Park Square, Luton, UK; 3 Institute for Health Research, University of Bedfordshire, Putteridge Bury, Hitchin Road, Luton, UK

**Keywords:** Health service access, Primary health care, Rurality, Demography

## Abstract

**Background:**

The Kingdom of Saudi Arabia (KSA) has seen an increase in chronic diseases. International evidence suggests that early intervention is the best approach to reduce the burden of chronic disease. However, the limited research available suggests that health care access remains unequal, with rural populations having the poorest access to and utilisation of primary health care centres and, consequently, the poorest health outcomes. This study aimed to examine the factors influencing the access to and utilisation of primary health care centres in urban and rural areas of Riyadh province of the KSA.

**Methods:**

A questionnaire survey was carried out to identify the barriers and enablers to accessing PHCS in rural (*n* = 5) and urban (*n* = 5) areas of Riyadh province, selected on the classification of the population density of the governorates. An adapted version of the NHS National Survey Programme was administered that included 50 questions over 11 sections that assessed a wide range of factors related to respondent’s access and experience of the PHCS. A total of 935 responses were obtained with 52.9% (*n* = 495) from urban areas and the remaining 47.1% (*n* = 440) from rural areas of Riyadh province.

**Results:**

This study highlights that there are high levels of satisfaction among patients among all PHCS. In relation to differences between urban and rural respondents, the findings indicated that there were significant variations in relation to: education level, monthly income, medical investigations, receiving blood tests on time, extra opening hours, distance, cleanliness and health prevention. Core barriers for rural patients related to the distance to reach PHCS, cleanliness of the PHCS, receiving health prevention and promotion services, which should serve to improve health outcomes.

**Conclusions:**

This study highlighted important differences in access to and utilisation of PHCS between urban and rural populations in Riyadh province in the KSA. These findings have implications for policy and planning of PHCCs and reducing inequalities in health care between rural and urban populations and contributing to a reduction in the chronic disease burden in Riyadh province.

**Electronic supplementary material:**

The online version of this article (doi:10.1186/s12913-017-1983-z) contains supplementary material, which is available to authorized users.

## Background

One of the core aims of the Sustainable Development Goals (SDG’s) is to provide all people throughout the world with equal, unbiased access to and ensure measures are in place to enable utilisation of basic Primary Health Care Services (PHCS) [1]. International evidence continues to demonstrate the fundamental role PHCS plays in improving population health through the reduction of morbidity and all-cause mortality [2, 3]. The impact of globalisation alongside the progression of developing and middle-income countries demographic and epidemiological transition has resulted in a rise in chronic disease [4]. It is reported that the Middle East and North African regions are now shown to have the highest regional prevalence of chronic diseases for 2011, (after age standardisation to the world population) [5, 6]. Consequently, tackling the rising chronic disease burden alongside the associated cost to the national health care systems [7, 8] represents a central agenda for policymakers when addressing changes to PHCS [9].

The Kingdom of Saudi Arabia (KSA) represents a middle Eastern country which has seen an increased chronic disease burden [10]. Current evidence has indicated that KSA has the 7^th^ highest rate of Diabetes Mellitus (DM) in the world [11, 12] alongside markedly increased rates of hypertension and coronary heart disease [13, 14]. This has, consequently, led to increased health costs to the government. For example, the current cost of diabetes in estimated at 17 billion Riyals [10] which is expected to increase to 43 billion Riyals [11].

The existing evidence base (the majority of which is based on evidence from the developed world) shows that early intervention has proven to be an effective strategy for reducing the incidence of chronic diseases and the difficulties, including the costs, associated with treatment of such diseases at the later stages of the conditions [15, 16]. Internationally, research suggests that access to and utilisation of PHCS can been unequal in countries between urban and rural (and nomadic) populations, with the latter having the poorest access to and utilisation of PHCS [10]. Rural (and nomadic) populations are also the most deprived groups within the KSA population [17, 18].

There is a paucity of evidence in comparing access to and utilisation of PHCS between urban and rural populations. By understanding the barriers and enablers to accessing PHCS in rural and urban areas in Riyadh province, KSA, this study will contribute towards reducing inequalities in access to and utilisation of PHCS. The objective of this study was to identify barriers and enablers in relation to access to and utilisation of PHCS among a sample of patients attending PHCS in rural and urban areas of Riyadh province.

## Methods

### Setting

The Riyadh province of Saudi Arabia was selected as the location for this study. The Riyadh province consists of twenty governorates (areas, districts or city). The twenty governorates in Riyadh province are not classified as either urban or rural based on any officially published statistics/record. Hence, it was proposed that the top quartile governorates will be classified as urban and the lower quartile as rural based on the population density [19] for the purposes of this study. The population density of each governorate was calculated by dividing the total population by the area of the corresponding governorate and the governorates were then arranged in a descending order of catchment population density to identify the ‘urban’ (top quartile) and the ‘rural’ (bottom quartile) governorates. Table 1 below presents the figures for the catchment population density for the twenty governorates in the Riyadh province.

**Table 1:** Catchment population density of Riyadh province of Saudi Arabia (MOI)

Governorate	Area/km^2^	Population	Catchment population density/km^2^	Rural/Urban
Alriyad	1800	5188286	2882.38	Urban
Al-Deri’yya	2020	73668	36.47	
Al-Kharj	19790	376325	19.02	
Al-Zulfi	5540	69294	12.51	
Dharma	2060	24429	11.86	
Al-Muzahmeya	3580	39865	11.14	Semi urban
Hraymla	1480	15324	10.35	
Shaqra	4110	40541	9.86	
Al-Dwadmy	30580	217305	7.11	
HotatBaniTameem	7350	43300	5.89	
Al-Ghat	2690	14642	5.44	
Al-Majma’ah	30000	133285	4.44	
Thadig	5600	17165	3.07	
Afeef	26810	77978	2.91	
Al-Quway’iyah	50580	126161	2.49	
Al-Hareeq	6790	14750	2.17	Rural
Wadi Al-Dawaser	48900	106152	2.17	
Rammah	15900	28055	1.76	
Al-Aflaj	54120	68201	1.26	
Al-Saleel	42420	36383	0.86	

Five rural and five urban Primary Health Care Centres (PHCC) in the Riyadh province were selected based on the classification of the population density of the governorates as discussed above (see Table 1). The selection of the PHCCs in these selected rural and urban governorates for the purpose of this study (data collection sites) was based on the Ministry of Health classification of services provided by the PHCCs. The Ministry of Health classifies its PHCCs based on the range of services provided. MOH classifies PHCCs that have a laboratory, dentistry and residential facilities for the GP and a nurse working at the PHCC with the identifier B3. After reviewing the Ministry of Health classifications for the PHCCs, it was observed that the most numerous categories of PHCSs were B3. To ensure like to like comparison (in terms of services offered) between rural and urban governorates, the B3 PHCCs serving the largest population in each governorate were identified as the PHC sites for inclusion in this study (Table 2).

**Table 2:** The PHCCs selected for the study (data collection sites) showing population density

Region	Name of PHCC	Population density	Governorate	Category
Rural	H	3980	Alaflaj	B3
	F	2599	Alhareeq	B3
	G	11495	WadiAldawasir	B3
	I	6614	Alsaleel	B3
	C	1033	Rimah	B3
Urban	A	2077	Darmaa	B3
	E	10536	Alzulfi	B3
	B	6065	Al-Deri’yya	B3
	D	8000	Riyadh	B3
	J	11368	Al-Kharj	B3

### Participants and methods

Participants were eligible to take part in the present study if they were aged 18 years or older, attended one of the recruiting PHCCs, were a Saudi resident and could consent. The sample size was calculated by using the following formulae **(1)n = Z** ^**2**^_**α**_ **P(1-P)/d** ^**2**^, where n = required sample size, Z_α_ = 1.96 (standard normal deviation), **P** = proportion of patients having access to and utilising the PHC and d = precision of estimate. Considering that 50% of patients have access to and are utilising their PHCCs both at urban and rural regions of Riyadh province, with a precision of ±5% and at 5% level of significance, 384 patients each were required at urban and rural PHCCs. The calculated sample size was 384 (*n* = (1.96)^2^ × (0.50 (1–0.50) / (0.05)^2^ = 384.16). With an anticipated 15% non-response and incomplete responses from the patients, the required sample size increased by 56. This represented a final target sample size of 440 from each urban and rural PHCCs, a total of 880 patients from the selected urban and rural PHCCs of Riyadh province (88 patients from each PHCC).

#### Data collection

A questionnaire survey was carried out to identify the barriers and enablers to accessing PHCS in rural and urban areas of Riyadh province. An adapted version of the NHS National Survey Programme: Primary Care Trust Question bank 2008 v6 dated 27th November 2007 was used. Permission to use and adapt the questionnaire was obtained from the Care Quality Commission in the UK [20–25]. The original questionnaire was a validated measuring instrument and included 123 questions in 15 sections covering information on local PHCS in the UK. The adapted final questionnaire used for this study included 50 questions over 11 sections. These sections included: (1) making an appointment, (2) visiting the PHCC, (3) seeing a doctor, (4) medicines, (5) tests, (6) referrals, (7) seeing another professional from a PHCC, (8) satisfaction of PHCC, (9) dental care and (10) health promotion alongside (11) personal socio-demographic information (see Additional file 1).

An Arabic version of the questionnaire, information sheet and consent form were developed. These were back translated by the lead researcher (GA) from UK English to Arabic and were piloted for interpretation and accuracy with Saudi postgraduate students studying at the University of Bedfordshire (*N* = 20). Minimal changes were needed. Modifications mostly related to formatting, i.e. increase of font size and improved presentation of information. In some cases, the Arabic translation used a very high level of Arabic grammar and pilot participants suggested that more ‘every day’ Arabic would be more appropriate to ensure that respondents fully understood the questionnaire.

All patients were recruited at each of the PHCC’s. To facilitate recruitment, practice managers at each of the PHCCs were sent a letter from the Ministry of Health, introducing the study. This was followed up by a phone call from a male research assistant with a date and time to set up a face-to-face meeting with the PHCC practice managers to discuss the research, enlist support to access patients and complete the questionnaire survey.

There were two phases of data collection. Phase one took place across a 3-month period (1 January 2014–31 March, 2014). In phase one PHCC nurses administered the questionnaires through 1–1 interviews with the patients in Arabic.

The lead researcher (GA) with the support of a male researcher provided a training support session with each PHCC practice team, which included practice managers and nursing staff who would administer the questionnaire. During this session, the lead researcher (GA) gave the team a protocol and recruitment packs that provided information on the patient eligibility criteria and the recruitment process. The questionnaires were discussed in detail alongside information on how they should be completed and what support nursing staff should provide. During the recruitment phase, a male research assistant communicated with the PHCC weekly to ascertain recruitment and engagement. Concerns were quickly identified that related to the resources needed for nurses to administer the questionnaires, with only 438 questionnaires completed during this period.

As such, a second phase was built in to the data collection process to facilitate higher recruitment. This phase was conducted across a 2-month period (1st May 2014–1st July 2014). The lead researcher (GS) and a male research assistant, who both had extensive experience in interviewing and administering questionnaires, visited each PHCC to recruit participants. The same recruitment protocol was applied with all questionnaires administered via 1–1 interviews in Arabic. This phase led to the collection of an additional 538 questionnaires. Overall, there were a total of 935 questionnaires collected from both recruitment phases. Table 3 provides the number of questionnaires handed out at each of the phases of the data collection, returned, excluded and the response rate for each PHCC.

**Table 3:** Number of questionnaire collected in the two phases of data collection with response rates by PHCC

PHCC Name	Classification (Urban/Rural)	Questionnaires collected (N) (phase one)	Questionnaires collected (N) (phase one)	Total Questionnaires	Questionnaires excluded (N)	Non-Response (N)	Questionnaires completed (N)	Response rate
(E)	Urban	22	78	109	6	3	94	86.24%
(A)	Urban	70	36	110	0	4	106	96.36%
(C)	Rural	75	25	105	3	2	97	92.38%
(D)	Urban	7	79	91	5	0	81	89.01%
(B)	Urban	45	70	125	7	3	108	86.40%
(J)	Urban	40	64	108	2	2	102	94.44%
(F)	Rural	40	55	106	10	1	85	80.19%
(H)	Rural	0	84	84	0	0	84	100.00%
(I)	Rural	62	22	89	5	0	79	88.76%
(G)	Rural	77	25	105	3	0	99	94.29%
Total	--------	438	538	1032	41	15	935	90.60%

### Statistical analysis

Categorical variables for all explanatory variables were calculated. Chi-square Goodness of Fit analyses were completed for frequencies with adjusted standardised residuals (ASR)’s that were calculated to indicate the importance of the cell to the ultimate chi-square value, which considers the overall sample size. This was particularly important given the varying counts by uptake rate across groups. Therefore, when reporting the results, the ASR values were used to indicate significance, i.e. ASR values of 3.09 (*p* < .001), 2.6 (*p* < .01) and 2 (*p* < .05) will signify significance, with anything below 2 deemed non-significant (*p* > .05). All statistical tests were completed using IBM SPSS for Windows, Version 22 [26]; two-tailed significance was assumed at *p* < 0.05.

### Ethics, consent and permissions

Ethical approval for this study was obtained from the University of Bedfordshire ethics committee and the KSA Ministry of Health.

## Results

A total of 935 responses were obtained with 52.9% of patient respondents from urban areas and the remaining 47.1% from rural areas. Results are presented in Table 4.

**Table 4:** Chi-square comparison of respondents by urban and rural location

		Rural			Urban				
		N	%	ASR	N	%	ASR	*X* ^2^	Sig
Socio-demographic characteristics									
Gender	Male	208	47.3	2.4	195	39.4	−2.4	5.9	**
	Female	232	52.7	−2.4	300	60.6	2.4		
Age	<20	25	5.7	0.6	24	4.8	−0.6	10.66	*
	21-30	141	32	−1.6	184	37.2	1.6		
	31-40	127	28.9	0.8	131	26.5	−0.8		
	41-50	95	21.6	1.5	87	17.6	−1.5		
	51-60	38	8.6	0.8	36	7.3	−1.5		
	60 year.+	14	3.2	−2.4	33	6.7	2.4		
Education level	0-16 years.	77	17.5	1.7	67	13.5	−1.7	13.26	**
	17-18 years.	106	24.1	−3.2	166	33.5	3.2		
	19 years+	222	50.5	2.3	213	43	−2.3		
	Still in education	35	8	−1	49	9.9	1		
Current monthly income	SAR 3,000 or less	168	38.2	−3.9	252	50.9	3.9	18.64	***
	SAR 3–8,000	240	54.5	2.7	226	45.7	−2.7		
	SAR 8–15,000	32	7.3	2.6	17	3.4	−2.6		
Health status									
Perceived health status	Excellent	58	13.2	0	65	13.1	0	1.84	*NS*
	Very good	233	53	−0.4	269	54.3	0.4		
	Good	145	33	0.7	152	30.7	−0.7		
	Fair	4	0.9	−1.2	9	1.8	1.2		
Prescribed medication	Yes	209	47.5	0.4	229	46.3	−0.4	0.14	*NS*
	No	231	52.5	−0.4	266	53.7	0.4		
Use of services									
Made apt. with doctor	Yes	0	0	−0.9	1	0.2	0.9	0.89	*NS*
	No	440	100	0.9	494	99.8	0.9		
Referral to specialist	Yes	265	60.2	1.8	270	54.5	−1.8	3.07	*NS*
	No	175	39.8	−1.8	225	45.5	1.8		
Medical investigations	No response	196	44.5	−6.1	319	64.4	6.1	64.69	***
	Yes	119	27	0.2	131	26.5	−0.2		
	No	116	26.4	7.4	41	8.3	−7.4		
	Do not remember	9	2	1.6	4	0.8	−1.6		
Organisational factors									
See doctor on time at apt.	Not at all	440	100	0.9	494	0.2	−0.9	0.89	*NS*
	Seen without apt.	0	0	−0.9	1	99.8	0.9		
Received blood results on time	No response	321	73	−0.2	364	73.5	0.2	6.33	*NS*
	Yes on time	84	19.1	−1	107	21.6	1		
	Later expected	34	7.7	2.3	21	4.2	−2.3		
	Still waiting	1	0.2	−0.9	3	0.6	0.9		
Opening hours	Yes often	30	6.8	0	34	6.9	0	0.66	*NS*
	Yes sometimes	125	28.4	0.8	129	26.1	−0.8		
	No	285	64.8	−0.7	332	67.1	0.7		
Extra opening times	No extra hours	234	53.2	3.3	210	42.4	−3.3	28.75	***
	Early mornings	11	2.5	0.5	10	2	−0.5		
	Evenings	103	23.4	−4.7	187	37.8	4.7		
	Saturdays	90	20.5	1.4	84	17	−1.4		
	Fridays	2	0.5	1.5	0	0	−1.5		
	No response	0	0	−1.9	4	0.8	1.9		
Extra opening days	No response	0	0	−1.9	4	0.8	1.9	17.67	***
	One day per week	124	28.2	−1.8	166	33.5	1.8		
	2-3 days per week	126	28.6	−0.3	146	29.5	0.3		
	4-5 days per week	57	13	3.6	30	6.1	−3.6		
	Don’t know	133	30.2	0	149	30.1	0		
Distance to PCC	Yes	123	28	5.7	64	12.9	−5.7	32.87	***
	No	317	72	−5.7	431	87.1	5.7		
Cleanliness of PCC	Very clean	245	55.7	−4	339	68.5	4	42.43	***
	Fairly clean	157	35.7	1.5	153	30.9	−1.5		
	Not very clean	24	5.5	4.4	3	0.6	−4.4		
	Not at all clean	12	2.7	3.7	0	0	−3.7		
	Unable to say	2	0.5	1.5	0	0	−1.5		
Mobility within PCC	Very easy	408	92.7	−3.1	481	97.2	3.1	12.16	**
	Fairly easy	31	7	3.2	13	2.6	−3.2		
	Not at all easy	0	0	−0.9	1	0.2	0.9		
	Unable to say	1	0.2	1.1	0	0	−1.1		
Help understanding Arabic	No response	1	0.2	−1.2	4	0.8	1.2	1.48	*NS*
	Yes	1	0.2	0.1	1	0.2	−0.1		
	No	438	99.5	1	490	99	−1		
Financial factors									
Pay for prescribed medicine/s	Yes	14	3.2	−1.1	23	4.6	1.1	1.31	*NS*
	No	426	96.8	1.1	472	95.4	−1.1		
Doctor patient communication									
Doctor listened carefully	Definitely	394	89.5	1.7	425	85.9	−1.7	2.91	*NS*
	To some extent	46	10.5	−1.7	70	14.1	1.7		
Enough time to discuss health	Definitely	385	87.5	1.5	416	84	−1.5	2.36	*NS*
	To some extent	50	11.4	−1.5	73	14.7	1.5		
	No	5	1.1	−0.1	6	1.2	0.1		
Treated with dignity and respect	Yes all of the time	440	100	2.3	489	98.8	−2.3	5.37	**
	Some of the time	0	0	−2.3	6	1.2	2.3		
Provided answers for questions	Yes definitely	370	84.1	0.8	406	82	−0.8	6.69	*NS*
	Yes to some extent	51	11.6	−1.5	74	14.9	1.5		
	No	4	0.9	0.2	4	0.8	−0.2		
	Did not need to	11	2.5	0.3	11	2.2	−0.3		
	No opportunity	4	0.9	2.1	0		−2.1		
Treatment explained & understood	Yes definitely	332	75.5	−0.8	384	77.6	0.8	18.5	***
	Yes to some extent	35	8	−2.5	64	12.9	2.5		
	No	3	0.7	−0.8	6	1.2	0.8		
	Did not want	32	7.3	3	15	3	−3		
	Not needed	38	8.6	2	26	5.3	−2		
Results explained & understood	Yes definitely	112	25.5	0.1	124	25.1	−0.1	4.95	*NS*
	Yes to some extent	7	1.6	1.1	4	0.8	−1.1		
	No response	321	73	−0.2	3	0.6	0.2		
	Still waiting	0	0	−1.6	364	7.35	1.6		
Health prevention and promotion									
Blood sugars checked at PCC	Yes	308	70	2.8	303	61.2	−2.8	7.96	**
	No	129	29.3	−2.8	188	38	2.8		
	Not sure	3	0.7	−0.2	4	0.8	0.2		
Received advice (weight)	Yes lose weight	207	47	−1.1	250	50.5	1.1	5.05	*NS*
	Yes stay the same	79	18	0.5	83	16.8	−0.5		
	Yes gain weight	21	4.8	−1	31	6.3	1		
	No like advice	71	16.1	2	58	11.7	−2		
	No advice wanted	62	14.1	−0.3	73	14.7	0.3		
Received advice (healthy eating)	Yes definitely	143	32.5	−2.1	193	39	2.1	21.82	***
	Yes to some extent	94	21.4	1	93	18.8	−1		
	Would like advice	131	29.8	4	92	18.6	−4		
	No advice wanted	72	16.4	−2.8	117	23.6	2.8		
Satisfaction									
Satisfaction of using PCC	Yes completely	388	88.2	0.8	428	86,5	−0.8	2.98	*NS*
	Yes to some extent	52	11.8	−0.5	64	12.9	0.5		
	No	0	0	−1.6	3	0.6	1.6		

### Socio-demographic characteristics

#### Gender and age

There was a total of 43.1% male and 56.1% female respondents. Chi-square analysis revealed that distribution was not equally distributed across the total sample by gender and region (*X* ^2^ = (2, *N* = 935) = 5.90, *p* < .01). Findings confirmed there were significantly more males from regions identified as ‘rural’ (ASR 2.4; p < .05) with significantly more females from regions identified as ‘urban’ (ASR 2.4; *p* < .05).

In relation to age distribution, chi-square analysis revealed that the distribution of age of respondents was not equally represented across both urban and rural regions (*X* ^2^ = (6, *N* = 935) = 10.66, *p* < .05). There were significantly fewer ‘older’ respondents (60 years+) from rural regions (ASR −2.4; *p* < .05) compared to urban.

### Education and income

Respondents from rural regions were more likely to have a higher level of education compared to those from urban regions (*X* ^2^ = (4, *N* = 935) = 13.26, *p* < .01). Specifically, those residing in urban areas were significantly more likely to have left education at 17–18 years old (ASR 3.2; *p* < .001) compared to rural areas where respondents were more likely to have left education at 19 years and older (ASR 2.3; *p* < .05). Furthermore, those from rural areas were significantly more likely to earn more income compared to those from urban areas (*X* ^2^ = (3, *N* = 935) = 18.64, *p* < .001). Chi square analysis revealed that those residing in rural areas were significantly more likely to earn SAR 3000–15,000 (*p* < .01) compared to those from urban areas who were significantly more likely to earn SAR 3000 or less (ASR 3.9; *p* < .001).

### Health status

There was no association by region (urban vs. rural) and health status (*X* ^2^ = (4, *N* = 935) = 1.84, *p* > .05). The majority of respondents rated their health as either very good (rural; 53%, urban; 54.3%) or good (rural; 33%, urban; 30.7%) with only a minority rating their health as poor.

### Use of services

There was no significant relationship between the region someone resides in (urban vs. rural) and seeing a doctor (*X* ^2^ = (2, *N* = 935) = 0.89, *p* > .05) with most respondents stating that they have not had an appointment with their doctor in the past 12 months (rural; 100%, urban; 99.8%). Likewise, there was no significant relationship between the region someone resides in (urban vs. rural) and being referred to a specialist (*X* ^2^ = (2, *N* = 935) = 3.07, *p* > .05) with all respondents stating that they have not been referred to a specialist in the past 12 months. In relation to medical investigations, there was a significant relationship between the region someone resides in (urban vs. rural) and having a blood test (*X* ^2^ = (3, *N* = 935) = 7.96, *p* > .01). The findings confirmed that respondents from rural regions were significantly more likely to have a blood test (ASR 2.8; *p* < .01) compared to those from urban regions (ASR −2.8; *p* < .01).

### Organisational factors

There was no significant relationship between the region someone resides in (urban vs. rural) and seeing their GP on time (*X* ^2^ = (2, *N* = 935) = 0.89, *p* > .05) with nearly all the respondents stating that they did not have to wait at all to see their doctor. Moreover, there was no significant relationship between the region someone resides in (urban vs. rural) and receiving blood test results on time (*X* ^2^ = (4, *N* = 935) = 6.33, *p* > .05).

There was no significant relationship between the region someone resides in (urban vs. rural) and if clinic hours negatively impacted on respondents seeing their doctor (*X* ^2^ = (3, *N* = 935) = 0.66, *p* > .05), with most respondents stating that opening hours was not an issue. There was a significant relationship between the region someone resides in (urban vs. rural) and wanting extra opening days (*X* ^2^ = (5, *N* = 935) = 17.67, *p* < .001) and times (*X* ^2^ = (6, *N* = 935) = 28.75, *p* < .001). The findings confirmed that respondents from urban regions were significantly more likely to want the centre to open early mornings (ASR 4.7; *p* < .001), with those from rural regions most likely to want the centre to open for extra days (ASR 3.6, *p* < .001).

In relation to the distance from patients’ residence to the primary care centre, there was a significant relationship between the region someone resides in (urban vs. rural) and distance posing an issue for attending the primary care centre (*X* ^2^ = (2, *N* = 935) = 32.87, *p* < .001). These findings suggested that distance was significantly more likely to present a problem to those residing in rural regions (ASR 5.7, *p* < .001) compared to those from urban regions (ASR −5.7, *p* < .001).

There was a significant relationship between the region someone resides in (urban vs. rural) and the cleanliness of the PCC (*X* ^2^ = (5, *N* = 935) = 42.43, *p* < .001) and ease of moving around with mobility (*X* ^2^ = (4, *N* = 935) = 12.16, *p* < .01). Respondents from rural regions were significantly more likely to state that the PCC was not very clean (ASR 4.4, *p* < .001) and not at all clean (ASR 3.7, *p* < .001) compared to those from urban regions who were significantly more likely to state the PCC is very clean (ASR 4, *p* < .001). Mobility appeared to be an issue for those who resided in a rural region. For example, significantly more people from urban regions stating it is very easy to get around (ASR 3.1, *p* < .001) compared to those from regions areas who were more likely to state it was only ‘fairly easy’ (ASR 3.1, *p* < .001).

There was no significant relationship between the region someone resides in (urban vs. rural) and help understanding Arabic (*X* ^2^ = (3, *N* = 935) = 1.48, *p* > .05), with most respondents stating that understanding Arabic was not an issue.

### Financial

Respondents were asked if they have had to pay for prescribed medicines in the past 12 months. Findings confirmed there was no significant relationship between the region someone resides in (urban vs. rural) and payment for prescriptions (*X* ^2^ = (2, *N* = 935) = 1.31, *p* > .05), with many respondents stating that they have not had to pay for medicines.

### Service provider-patient communication

There was no significant relationship between the region someone resides in (urban vs. rural) and whether the respondent’s doctor listened carefully (*X* ^2^ = (2, *N* = 935) = 2.91, *p* > .05), provided enough time to discuss health issues (*X* ^2^ = (3, *N* = 935) = 2.36, *p* > .05) and provided answers for questions (*X* ^2^ = (2, *N* = 935) = 6.69, *p* > .05) and satisfactorily explained investigative test results (*X* ^2^ = (4, *N* = 935) = 4.95, *p* > .05). Most respondents viewed the doctor favourably across all factors.

However, there were significant differences noted for ‘being treated with dignity and respect’ (*X* ^2^ = (2, *N* = 935) = 5.37, *p* < .001) and ‘treatment explained and understood’ (*X* ^2^ = (5, *N* = 935) = 18.5, *p* < .01). For example, respondents from rural areas felt that their doctor treated them with dignity and respect ‘all of the time’ (ASR 2.3; *p* < .01) compared with urban respondents who were significantly more likely to state ‘only some of the time’ (ASR 2.3; *p* < .01). However, in relation to communication relating to treatment, urban respondents were more significantly likely to state treatment was explained well and was well understood (ASR 2.5 *p* < .01) compared to those from the rural areas.

### Service provision

There was no significant relationship between the region someone resides in (urban vs. rural) and receiving advice related to weight (*X* ^2^ = (2, *N* = 935) = 5.05, *p* > .05). However, there were significant differences found for blood sugars being checked (*X* ^2^ = (3, *N* = 935) = 7.96, *p* < .01) and receiving advice relating to healthy eating (*X* ^2^ = (4, *N* = 935) = 21.82, *p* < .001). The findings confirmed respondents from rural regions were more likely to have their blood sugars checked (ASR 2.8, *p* < .01). However, respondents from urban regions were significantly more likely to ‘definitely’ receive advice relating to healthy eating (ASR 2.1; *p* < .05), with those from rural regions less likely to receive advice but significantly more likely to want advice (ASR 4; *p* < .001).

### Overall satisfaction

Respondents were asked to rate their level of satisfaction of using the PCC. There was no significant relationship between the region someone resides in (urban vs. rural) and satisfaction (*X* ^2^ = (3, *N* = 935) = 2.98, *p* > .05), with the majority of respondents completely satisfied.

## Discussion

### Socio-demographic characteristics

There was higher female participation compared to males, which could be reflective of more general patterns of health care utilisation [27, 28]. However, research that has explored health service use in Islamic societies has shown that often females have lower rates of healthcare utilisation [29]. This is often because they remain dependent on men to make decisions about healthcare, with women not normally allowed out to visit a health facility or health care provider alone [30]. The higher participation of females in non-Islamic societies may also be related to the presence of a female administering the questionnaire during recruitment; an essential consideration when conducting research with women in the Saudi Traditional Islamic segregated context [31, 32].

Socio-economic factors, such as income, education and employment, are key enabling characteristics for accessing health care, particularly in terms of the ability to pay for health insurance [33–35]. The monthly income of patient respondents ranged from less than 3000–15,000 Riyals per month, with highest income rates shown for 8000 Riyals and less. This is representative of the average monthly incomes in the KSA [36]. Interestingly, respondents from rural regions earned significantly more (3–15,000 Riyals) compared to urban regions (<3000 Riyals). Furthermore, respondents from rural regions were more likely to have a higher level of education and to have left education later compared to those from urban regions. Traditionally, urban regions and cities were viewed as focal points of economic growth, employment and innovation, all factors shown to be indicative of good general health and wellbeing [37]. However, it is now strongly argued that the proportion of urban poor in developing countries worldwide is increasing faster that of the overall rate of urban population growth [38]. For example, 30% of the urban population of the Middle East and North Africa live in Slums [39].

### Health Status

The majority of respondents from both urban and rural locations rated their health as good and very good irrespective of what region they were from. This is an interesting finding given that 47.5% (*n* = 209) of rural and 46.3% (*n* = 229) of urban patients are taking prescribed medication. Health care research has shown that beliefs and understandings surrounding a patient’s illness in culturally diverse groups are not only a core facilitator of health service uptake [40, 41] but also medication adherence [42]. Consequently, future research should examine the illness beliefs of patients in relation to their health status, co-morbidities and medication to determine what processes are related to help-seeking behaviour and, consequently, access.

### Use of services

The majority of respondents from both urban and rural regions stated that they have not made an appointment with a doctor in the past 12 months. Whilst this may relate to recall bias, this does not correspond with other findings. For example, many patients were referred to a specialist and likewise had medical investigations during the same period. Patients from rural areas were significantly less likely to have had a medical investigation in the past 12 months. Further, whilst the majority of respondents from urban areas who had a medical investigation stated they received test results on time, for rural participants this was significantly later than expected. International evidence has suggested that provision of medical care is poorer in rural communities [43] particularly those in developing countries, which often is related to lower proportions of healthcare professions [44] and reduced access to medical resources [45].

### Organisational factors

Whilst it may have been hypothesized that rural patients would want extra opening hours compared to their urban counterparts, this finding was not supported. For example, urban respondents were shown to want increased opening hours particularly in the evenings, with rural respondents significantly more likely to state that they did not want extra opening hours. This may be related to the economic growth and the increased levels of employment found in the more urbanised provinces of KSA [46]. Further, this finding may be related to the international legislative commitments that have centred on women’s welfare and increasing their societal and economic role [47, 48]. For example, recent evidence has shown that the number of women in employment in Saudi Arabia quadrupled, from 48,000 in 2009 to over 200,000 in 2012 [48]. Differences are found in urban and rural communities, with rural mothers less likely to be employed and more likely to engage in household chores and parental activities [49, 50]. Inconvenient clinic times for PHCC remains a core barrier to accessing health services [51–54], more flexible opening times for urban respondents should serve to improve health outcomes and increase levels of patient satisfaction.

Distance from the patients’ residence was viewed as a barrier to accessing PHCCs for participants residing in rural areas but not from those residing in urban areas. There is an extensive evidence base that continues to suggest that distance from health facilities, often referred to as the ‘distance decay’ effect, has a detrimental impact on health care utilisation [55–57]. It is widely reported that the KSA population is reluctant to travel long distances to PHCCs because of the hot climate found in the summer months [56, 58]. Households within KSA have high rates of car ownership, so the availability of transportation to the PHCC was not considered an important factor that may intersect with distance from residence as a barrier to accessing the PHCC [59].

The majority of respondents stated it was very easy for them to move around inside the PHCC. This is particularly important in a segregated society like the KSA because, without clearly defined (and resourced) spaces for women and men, access to the PHCC would be a major barrier to accessing and utilising the PHCCs [60]. However, in relation to cleanliness, there were marked differences. For example, rural respondents were significantly more likely to state that the cleanliness was ‘not at all clean’ and ‘not very clean,’ with urban respondents significantly more likely to state their PHCC was ‘very clean’. This has been found in other rural areas of middle income countries where standards are not always consistent [61]. A recent review by the World Health Organisation stated that primary health care centres have significantly lower water, sanitation and hygiene in middle income countries, although this is viewed as problematic for rural residents as often this is the only point of contact [62]. As such, national planning should remain key in improving services and sanitation to improve the delivery of routine services and to prevent and control infections.

However, it is also important to note that the rural sample was characterised by higher levels of income and education. Further, there were more men; therefore, this finding could be related to increased levels of expectations, particularly within in a society with a strong gender hierarchy among wealthier and better-educated men. Research has highlighted that acceptability and levels of satisfaction towards health services, particularly in Middle Eastern countries is higher among disadvantaged groups (women and the poor) compared to men and those with higher levels of income [63]. This, therefore, suggests that satisfaction of services may be variable and is dependent on the local context [64].

### Doctor patient communication

This study found that there was good overall communication between the doctor and patient respondents. The majority of patient respondents said that their doctor listened carefully, had enough time to discuss their medical problem and, if they had questions to ask the doctor, they got the answers and were treated with respect and dignity. These are some of the ideal features patients report as being important to creating positive relationships between the doctor (and other health care professionals) and patient [65–67]. However, there were significant differences found between patient respondents in urban and rural areas in relation to understanding the treatment or action that was explained, with fewer rural patient participants feeling that doctors explained reasons for any treatments. This may be related to levels of expectations as previously reported.

All the GPs included in this study were a non-Saudi national, which is the norm in KSA [68] (Egyptian, Pakistani, Sudanese and Tunisian), but spoke Arabic and were Muslim, which may be one reason for positive patient responses about seeing the doctor. None of the respondents said they needed help speaking Arabic. We can surmise, therefore, that having doctors who are able to communicate in the vernacular was an enabler for patients accessing and utilising the PHCC. The cultural competency of non-Saudi health professionals has been argued as impacting on patient satisfaction with HCS in the context of KSA, with calls for increased cultural awareness training for non-Saudi health care professional [68].

### Health prevention and promotion

All patient respondents said that they had their blood sugar levels measured and were given advice on weight and advice on healthy eating. Results also showed that patient respondents attending PHCC in urban areas were being given more advice on eating a healthy diet compared to patient respondents attending rural PHCCs. It is clear that the PHCCs are monitoring and addressing the health education needs of their patients related to chronic diseases, but increasing prevalence rates of chronic diseases in the KSA and in the Riyadh province suggest that more health education is needed to stem the epidemic of chronic disease in the KSA [69–72].

### Overall satisfaction with the PHCCs

Patient satisfaction has been used as an indication/measure of quality of care. Overall, patient participants were positive about their PHCCs and the majority said they went to the PHCC because the PHCC dealt with them in a satisfactory way. This research highlighted that patients had high levels of satisfaction with this finding consistently for both urban and rural patients. This finding supports existing evidence, which has reported high levels of patient satisfaction with PHCC’s in the KSA [71, 73, 74].

### Strengths and limitations

This study has provided some important information on the barriers and enablers to the access and utilisation of PHCS in the Riyadh province in the KSA. Nonetheless, there are several limitations to the study that are noteworthy. The lead researcher (GA) was working in a gender-segregated society where women are not permitted to access male spaces. The gender bureaucracy in the KSA meant that the success of this study, therefore, was heavily dependent on the support of a *mahram* who acted as the research assistant (*mahram)*. Consequently, GA was dependent on her *mahram* to support and negotiate access to research sites, practice managers and accessing male patients.

A further limitation related to the patient questionnaire. On some occasions, it was unclear if there was no answer because the question was not applicable or if this was a true missing variable. For example, section E (Test) and section K (Dental care) (see Additional file 1) did not ask patients if they had the relevant facilities in their PHCC. Therefore, if this section was missing, it may have been that the PHCC did not have the facility rather than a true missing variable. This may have impacted the validity of the findings.

## Conclusions

Our study demonstrated that there are important differences in access to and utilisation of PHCS between urban and rural populations. Further studies evaluating patient access, utilisation and experiences in other areas of the KSA will assist policy makers and service providers and provide insight into the required service provision. Our study highlights that there are high levels of satisfaction among patients regarding PHCS. However, distance to reach PHCS and extended opening hours are key issues to consider when commissioning PHCS for rural residents.

## Additional file

Additional file 1: Primary Health Care services questionnaire. (DOC 104 kb)

## Abbreviations

ASR: Adjusted standardised residual
DM: Diabetes mellitus
KSA: Kingdom of Saudi Arabia
PHCC: Primary Health Care Centres
PHCS: Primary Health Care Services
