# Supplementary material for: Access and utilisation of primary health care services comparing urban and rural areas of Riyadh Providence, Kingdom of Saudi Arabia
Source: BMC Health Serv Res. 2017 Feb 2;17:106. doi: 10.1186/s12913-017-1983-z (PMC5288856; doi:10.1186/s12913-017-1983-z)
Supplement: Additional file 1: — Primary Health Care services questionnaire. (DOC 104 kb) [file 12913_2017_1983_MOESM1_ESM.doc]

**Primary Health Care Services Questionnaire**

1. making an appointment

**Please answer these questions thinking about any health care EITHER for yourself OR for a child in your care**

1. Have you **made an appointment** with a doctor from your primary health care centre in the last 12 months?

1 🞏 Yes **🡺 Go to A2**

2 🞏 No **🡺 Go to B1**

Thinking about your LAST appointment or home visit …

1. The **last** time you saw a doctor from your primary health care centre, how long did you wait for an appointment?

1 🞏 I was seen without an appointment **🡺 Go to A5**

2 🞏 I was seen on the same working day **🡺 Go to A4**

3 🞏 I waited 1 or 2 working days **🡺 Go to A3**

4 🞏 I waited more than 2 working days

**🡺 Go to A3**

5 🞏 It was a pre-planned appointment or visit **🡺 Go to A4**

6 🞏 Can’t remember **🡺 Go to A4**

1. What was the main reason you waited? (**Tick ONE only**)

1 🞏 I wanted to see **my own choice** of doctor

2 🞏 I could not get an earlier appointment with **any** doctor at my health centre

3 🞏 It was **not convenient for me** to have an appointment at any earlier time

4 🞏 Another reason

1. How do you feel about the length of time you waited for an appointment with a doctor?

1 🞏 I was seen as soon as I thought was necessary

2 🞏 I should have been seen **a bit sooner**

3 🞏 I should have been seen **a lot sooner**

1. If you want to make a doctor’s appointment **3 or more working days in advance** does your primary health care centre allow you to do that?

1 🞏 Yes

2 🞏 No

3 🞏 Don’t know/ Not sure

1. visiting the primary HEALTH care CENTRE
2. Is the distance from your residence an issue in visiting your primary health care centre?

1 🞏 Yes

2 🞏 No

Thinking about your LAST visit to the health centre…

1. How long **after your appointment time** did you have to wait to be seen?

1 🞏 I did not have an appointment

2 🞏 Seen on time or early

3 🞏 Waited up to 15 minutes

4 🞏 Waited 16-30 minutes

5 🞏 Waited 31 minutes or longer

6 🞏 Can’t remember

1. SEEING A Doctor

***Thinking about the LAST TIME you saw a doctor from your health centre…***

1. Did the doctor **listen carefully** to what you had to say?

1 🞏 Yes, definitely

2 🞏 Yes, to some extent

3 🞏 No

1. Were you given **enough time** to discuss your health or medical problem with the doctor?

1 🞏 Yes, definitely

2 🞏 Yes, to some extent

3 🞏 No

4 🞏 I did not need to discuss anything

1. If you had **questions** to ask the doctor, did you get answers that you could understand?

1 🞏 Yes, definitely

2 🞏 Yes, to some extent

3 🞏 No

4 🞏 I did not need to ask any questions

5 🞏 I did not have an opportunity to ask questions

1. Did the doctor explain the reasons for any treatment or action in a way that you could understand?

1 🞏 Yes, completely

2 🞏 Yes, to some extent

3 🞏 No

4 🞏 I did not need an explanation

5 🞏 No treatment or action was needed

1. Did the doctor treat you with **respect and dignity**?

1 🞏 Yes, all of the time

2 🞏 Yes, some of the time

3 🞏 No

1. MEDICINES (e.g. tablets, ointment, oral contraceptives)

Thinking about the LAST time you had a new medicine prescribed for you …

1. Have you been taking any prescribed medicine(s) for 12 months or longer?

1 🞏 Yes

2 🞏 No

1. Did you have to pay for any prescribed medicine(s) for last 12 months?

1 🞏 Yes

2 🞏 No

1. In the last 12 months, have you asked a pharmacist for any advice on medicines?

1 🞏 Yes **🡺 Go to D4**

2 🞏 No **🡺 Go to D5**

1. Was the pharmacist’s advice helpful?

1 🞏 Yes, definitely

2 🞏 Yes, to some extent

3 🞏 No

4 🞏 Not sure

1. In the last 12 months, have you asked a traditional healer for any advice on medicines?

1 🞏 Yes **🡺 Go to D6**

2 🞏 No **🡺 Go to E1**

1. Was the traditional healer’s advice helpful?

1 🞏 Yes, definitely

2 🞏 Yes, to some extent

3 🞏 No

4 🞏 Not sure

1. TESTS
2. In the last 12 months, have you had any tests (e.g. blood tests, swabs, smear tests) carried out by **anyone** from your primary health care centre?

1 🞏 Yes **🡺 Go to E2**

2 🞏 No **🡺 Go to F1**

3 🞏 Can’t remember **🡺 Go to F1**

Thinking about your most recent test(s)….

1. Did you get your test results on time?

1 🞏 Yes, I got them on time or early

2 🞏 No, I got the results later than expected

3 🞏 I am still waiting for the results

4 🞏 I did not get the results at all

1. Did someone explain the results of the tests in a way you could understand?

1 🞏 Yes, definitely

2 🞏 Yes, to some extent

3 🞏 No

4 🞏 I am still waiting for the results

5 🞏 Not sure/ can’t remember

1. Referrals
2. **In the last 12 months**, has anyone at your primary health care centre referred you to a specialist (e.g. a hospital consultant)?

1 🞏 Yes

2 🞏 No

1. SEEING Another professional from this primary health care centre
2. Have you seen anyone else from your primary health care centre **other than a doctor** in the last 12 months?

1 🞏 Yes **🡺 Go to G2**

2 🞏 No **🡺 Go to J1**

1. The **last time** you saw someone other than a doctor from your primary health care centre, who did you see? (**Tick ONE only**)

1 🞏 A nurse practitioner

2 🞏 A midwife

3 🞏 A dentist

4 🞏 A health educator

5 🞏 Someone else

6 🞏 I was not sure who I saw

1. The **last** time you saw this person, how long did you wait for an appointment?

1 🞏 I was seen without an appointment

**🡺 Go to G6**

2 🞏 I was seen on the same working day **🡺 Go to G5**

3 🞏 I waited 1 working day **🡺 Go to G4**

4 🞏 I waited 2 working days **🡺 Go to G4**

5 🞏 It waited more than 2 working days

**🡺 Go to G4**

6 🞏 It was a pre-planned appointment or visit

**🡺 Go to G5**

7 🞏 Can’t remember **🡺 Go to G5**

1. What was the main reason you waited? (**Tick ONE only**)

1 🞏 I wanted to see **my own choice** of professional

2 🞏 I could not get an earlier appointment with **any** **other professional** at my health centre

3 🞏 It was **not convenient for me** to have an appointment at any earlier time

4 🞏 Another reason

1. How do you feel about the length of time you waited for an appointment with this person?

1 🞏 I was seen as soon as I thought was necessary

2 🞏 I should have been seen **a bit sooner**

3 🞏 I should have been seen **a lot sooner**

***Still thinking about the LAST TIME you saw someone other than a doctor from your health centre…***

1. Did that person explain the reasons for any treatment or action in a way that you could understand?

1 🞏 Yes, completely

2 🞏 Yes, to some extent

3 🞏 No

4 🞏 I did not need an explanation

5 🞏 No treatment or action was needed

1. Did that person treat you with **respect and dignity**?

1 🞏 Yes, all of the time

2 🞏 Yes, some of the time

3 🞏 No

1. Did you have **confidence and trust** in that person?

1 🞏 Yes, definitely

2 🞏 Yes, to some extent

3 🞏 No

1. Overall about your health centre
2. Was the main reason you went to your primary health care centre dealt with to your satisfaction?

1 🞏 Yes, completely

2 🞏 Yes, to some extent

3 🞏 No

1. In your opinion, how clean is the primary health care centre?

1 🞏 Very clean

2 🞏 Fairly clean

3 🞏 Not very clean

4 🞏 Not at all clean

5 🞏 Can’t say

1. How easy do you find it to move around inside the primary health care centre?

1 🞏 Very easy

2 🞏 Fairly easy

3 🞏 Not at all easy

4 🞏 Can’t say

1. In the last 12 months, have you ever been put off going to your primary health care centre because the opening times are inconvenient for you?

1 🞏 Yes, often

2 🞏 Yes, sometimes

3 🞏 No

1. If it were possible for your primary health care centre to open at additional times, which of these times would you most like it to be open? **(Tick ONE only)**

1 🞏 No extra hours

2 🞏 Early mornings (before 8am)

3 🞏 Evenings (after 6pm)

4 🞏 Saturdays

5 🞏 Fridays

1. If your primary health care centre were to be open either earlier in the morning or later in the evening, how many days a week would you want this to happen?

1 🞏 One day per week

2 🞏 Two or three days per week

3 🞏 Four or five days per week

4 🞏 Don’t know

1. If your primary health care centre were to be open extra hours **but** had to close for some of its normal hours to allow this, would this be acceptable to you?

1 🞏 Yes, completely

2 🞏 Yes, to some extent

3 🞏 No

1. Do you need any help understanding Arabic?

1 🞏 Yes **🡺 Go to J9**

2 🞏 No **🡺 Go to K1**

1. The **last** time you saw someone from your primary health care centre who did not speak your language, was there someone who could interpret for you?

1 🞏 Yes, a relative or friend

2 🞏 Yes, someone from the practice/health centre staff

3 🞏 No

1. dental care
2. Do you visit a dentist regularly (that is at least once every 2 years)?

1 🞏 Yes – at a Primary Health Care centre

2 🞏 Yes – privately

3 🞏 No

4 🞏 Don’t know

1. In the last 24 months, have you visited a dentist **at a primary health care centre**?

1 🞏 Yes

2 🞏 No

3 🞏 Not sure/ Can’t remember

**K3.** Overall, was the main reason for this visit

dealt with satisfactorily?

1 🞏 Yes, completely

2 🞏 Yes, to some extent

3 🞏 No

1. HEALTH PROMOTION
2. In the last 12 months have you had your **blood sugar levels** measured by anyone from your primary health care centre?

1 🞏 Yes

2 🞏 No

3 🞏 Not sure/ can’t remember

1. In the last 12 months, have you been given advice from your primary health care centre on your **weight**?

1 🞏 Yes – I was told I should try to lose weight

2 🞏 Yes – I was told I should try to stay the same weight

3 🞏 Yes – I was told I should try to gain weight

4 🞏 No, but I would have liked some advice

5 🞏 No, but I did not want any advice

1. In the last 12 months, have you been given advice or help from your primary health care centre on **eating a healthy diet**?

1 🞏 Yes, definitely

2 🞏 Yes, to some extent

3 🞏 No, but I would have liked help/advice

4 🞏 No, but I did not want any help/advice

1. About you
2. Are you male or female?

1 🞏 Male

2 🞏 Female

1. What was your **year** of birth?

| **(Please write in)** e.g. | 1 | 9 | 3 | 4 |
| --- | --- | --- | --- | --- |

|  |  |  |  |
| --- | --- | --- | --- |

*please write approximate year if not sure.

1. How old were you when you left full-time education?

1 🞏 16 years or less

2 🞏 17 or 18 years

3 🞏 19 years or over

4 🞏 Still in full-time education

1. What is your current monthly income

1 🞏 SAR 3000 or less

2 🞏 SAR 3000 to 8000

3 🞏 SAR 8000 to 15000

4 🞏 Over SAR 15000

**P5.** Overall, how would you rate your health during

the **past 4 weeks**?

1 🞏 Excellent

2 🞏 Very good

3 🞏 Good

4 🞏 Fair

5 🞏 Poor

6 🞏 Very pood
